# Identification of anti-SF3B1 autoantibody as a diagnostic marker in patients with hepatocellular carcinoma

**DOI:** 10.1186/s12967-018-1546-z

**Published:** 2018-06-28

**Authors:** Hai-Min Hwang, Chang-Kyu Heo, Hye Jung Lee, Sang-Seob Kwak, Won-Hee Lim, Jong-Shin Yoo, Dae-Yuel Yu, Kook Jin Lim, Jeong-Yoon Kim, Eun-Wie Cho

**Affiliations:** 1 Rare Disease Research Center, Korea Research Institute of Bioscience and Biotechnology, 125 Gwahak-ro, Yuseong-gu, Daejeon, 34141 South Korea; 2 Department of Microbiology and Molecular Biology, College of Bioscience and Biotechnology, Chungnam National University, 99 Daehak-ro, Yuseong-gu, Daejeon, 34134 South Korea; 3 Proteometech Inc., 1101 Wooree Venture Town, 466 Gangseo-ro, Gangseo-gu, Seoul, 07573 South Korea; 4 Graduate Program for Nanomedical Science, Yonsei University, 50 Yonsei-ro, Seodaemun-gu, Seoul, 03722 South Korea; 5 Department of Functional Genomics, University of Science and Technology, 125 Gwahak-ro, Yuseong-gu, Daejeon, 34141 South Korea; 6 Biomedical Omics Group, Korea Basic Science Institute, 162 YeonGuDanji-ro, Ochang-eup, Cheongju, Chungbuk 28119 South Korea; 7 Disease Model Research Laboratory, Korea Research Institute of Bioscience and Biotechnology, 125 Gwahak-ro, Yuseong-gu, Daejeon, 34141 South Korea

**Keywords:** Autoantibody biomarker, SF3B1, Hepatocellular carcinoma, Cyclic peptide epitope, Human serum ELISA

## Abstract

**Background:**

Tumor-associated (TA) autoantibodies, which are generated by the immune system upon the recognition of abnormal TA antigens, are promising biomarkers for the early detection of tumors. In order to detect autoantibody biomarkers effectively, antibody-specific epitopes in the diagnostic test should maintain the specific conformations that are as close as possible to those presenting in the body. However, when using patients’ serum as a source of TA autoantibodies the characterization of the autoantibody-specific epitope is not easy due to the limited amount of patient-derived serum.

**Methods:**

To overcome these limits, we constructed a B cell hybridoma pool derived from a hepatocellular carcinoma (HCC) model HBx-transgenic mouse and characterized autoantibodies derived from them as tumor biomarkers. Their target antigens were identified by mass spectrometry and the correlations with HCC were examined. With the assumption that TA autoantibodies generated in the tumor mouse model are induced in human cancer patients, the enzyme-linked immunosorbent assays (ELISA) based on the characteristics of mouse TA autoantibodies were developed for the detection of autoantibody biomarkers in human serum. To mimic natural antigenic structures, the specific epitopes against autoantibodies were screened from the phage display cyclic random heptapeptide library, and the streptavidin antigens fused with the specific epitopes were used as coating antigens.

**Results:**

In this study, one of HCC-associated autoantibodies derived from HBx-transgenic mouse, XC24, was characterized. Its target antigen was identified as splicing factor 3b subunit 1 (SF3B1) and the high expression of SF3B1 was confirmed in HCC tissues. The specific peptide epitopes against XC24 were selected and, among them, XC24p11 cyclic peptide (-CDATPPRLC-) was used as an epitope of anti-SF3B1 autoantibody ELISA. With this epitope, we could effectively distinguish between serum samples from HCC patients (n = 102) and healthy subjects (n = 85) with 73.53% sensitivity and 91.76% specificity (AUC = 0.8731). Moreover, the simultaneous detection of anti-XC24p11 epitope autoantibody and AFP enhanced the efficiency of HCC diagnosis with 87.25% sensitivity and 90.59% specificity (AUC = 0.9081).

**Conclusions:**

ELISA using XC24p11 peptide epitope that reacts against anti-SF3B1 autoantibody can be used as a novel test to enhance the diagnostic efficiency of HCC.

**Electronic supplementary material:**

The online version of this article (10.1186/s12967-018-1546-z) contains supplementary material, which is available to authorized users.

## Background

Cancer is a serious disease with a high mortality rate, which it is often diagnosed in the late stage of the disease. However, early diagnosis strategies can improve cancer outcomes by providing care at the earliest possible stage [1]. Currently, high-resolution imaging of cancer is possible [2], but biomarker detection methods using body fluids are still most suitable for routine verification of health information. Blood is especially useful for in vitro diagnostic tests because it circulates throughout all the organs in the body, loading their secretions or traces which can then be used as cancer biomarkers.

Most of the currently used clinical cancer biomarkers, including alpha-fetoprotein (AFP), are tissue-secreted antigens that can be detected by immunological methods [3]. In addition, many intracellular protein antigens derived from tumor tissues have been suggested as serum biomarkers [4]. Recently, the components of extracellular vesicles from body fluids, especially cell-free DNA or microRNAs, also have been suggested as cancer biomarkers [5, 6]. However, the amount of any of these biological markers present in the blood depends on the size of the tumor, limiting its clinical use for early diagnosis.

TA autoantibodies are currently emerging as strong candidates for cancer biomarkers that reflect the existence of TA antigens [7, 8]. TA autoantibodies are produced by the self-immune system triggered by TA antigens even at low concentrations that cannot be detected by in vitro diagnostics, and biologically amplify the detectable signals for the corresponding antigens. On these grounds, TA autoantibodies are feasible biomarkers even in the early stage of tumorigenesis [7, 9]. Moreover, the antibodies persist in the circulation with half-lives typically up to 30 days and are more stable outside the body than other biomarkers, which is a great advantage as an in vitro testing biomarker [7]. In addition, the development of diagnostic tests for TA autoantibody biomarkers only require specific antigens because the quantification platforms are already used for common clinical use [7, 10]. However, despite all of these advantages, TA autoantibody biomarkers that are put to practical use are rare. In most of studies on TA autoantibody biomarker, patients’ serum has been used as a source of TA autoantibodies. However, the characterization of the autoantibody-specific epitope using patient-derived sera is not easy due to the limited amount of serum available.

HCC is the most common type of primary liver cancer in adults and is the most common cause of death in people with cirrhosis [11], and AFP is still considered to be the gold standard biomarker of HCC, although it has been challenged due to the lack of sensitivity [12, 13]. The hepatitis B virus X (HBx)-protein, encoded by the HBV X gene, is believed to play a vital role in the pathogenesis of HCC [14, 15]. With these backgrounds, HBx-transgenic (HBx-Tg) liver cancer model mouse was developed to simulate human HCC liver cancer, of which the HCC incidence is approximately 86% and shows similar characteristics to those of human HCC carcinogenesis [15–27]. In HBx-Tg mice, the autoantibodies related to liver cancer also can be induced as in HCC patients. On the assumption of the presence of TA autoantibodies in tumor model mouse, we collected splenocyte B cells from HBx-Tg mice with HCC and constructed a B cell hybridoma pool. One of TA autoantibodies secreted from HBx-tg mouse derived B cell hybridoma, designated as XC24, showed a specific response to human tumor cells including hepatoma, which was characterized in this study. Using purified monoclonal XC24 antibody, we identified its specific target antigen as splicing factor 3B subunit 1 (SF3B1). Also, we screened the conformational peptide epitope (-CDATPPRLC-) against XC24 from M13 phage display cyclic peptide (-CX_7_C-) library and suggested its application to human HCC diagnosis.

## Methods

### TA autoantibody, cell lines, tissues and human serum samples

The mouse B cell clones secreting a monoclonal autoantibody reactive to human hepatoma cells were selected from a B cell hybridoma pool constructed using hepatitis B virus X (HBx)-transgenic (tg) mice, as described previously [28]. The cancer cell lines were obtained from the American Type Culture Collection (Manassas, VA, USA). All cell lines originated from humans, except for HT22, which is a mouse hippocampal neuronal cell line. The cells were cultured in Dulbecco’s modified Eagle medium or RPMI-1640 (Thermo Scientific, Waltham, MA, USA) supplemented with 10% fetal bovine serum (Sigma-Aldrich, St Louis, MO, USA). Mouse liver or HCC tissues used for western blotting or immunohistochemistry were obtained from H*ras*12V- or HBx-tg mice provided by Disease Model Research Laboratory of KRIBB [15, 29]. Exosome-containing conditioned media were prepared from 70 to 80% confluent cancer cells, which were cultured for 48 h with exosome-free fetal bovine serum. Human HCC serum samples for this study were provided by the Ajou Human Bio-Resource Bank (AHBB; Suwon, Korea), a member of the National Biobank of Korea (Additional file 1: Table S1). Normal human serum samples (Non-HCC) were provided by the Korean Red Cross (Seoul, Korea). Serum samples were kept at − 70 °C until use.

### Identification of target antigen against XC24 TA autoantibody

XC24, an autoantibody secreted from one of our tumor model mouse B-cell clones, was analyzed in this study. The isotype of XC24 autoantibody was determined using a Pierce Rapid ELISA Mouse mAb Isotyping Kit (Thermo) as immunoglobulin (Ig) M. Identified XC24 autoantibody was purified from ascites fluid or hybridoma cell culture media using protein L-agarose (Thermo).

To examine the reactivity of XC24 autoantibody to human cancer cells, flow cytometric analysis and immunofluorescence microscopy were performed. For flow cytometric analysis, the suspended cells were fixed and permeabilized with BD cytoperm/cytofix solution (BD Bioscience, Franklin Lakes, NJ, USA), followed by an incubation with primary antibody solution and with goat anti-mouse IgG F(ab′)_2_-FITC. The stained cells were analyzed by FACScalibur (BD) and obtained data were analyzed using CellQuest software (BD). When determining whether the autoantibody-mimotope phage can compete with target cellular antigen for antibody binding, primary antibodies were pre-incubated with each phage at room temperature for 60 min and then used for staining.

To detect the XC24 antibody-specific antigen, western blot analysis of tumor cell lysates was performed. Cell lysates were prepared with NP40 lysis buffer [PBS (10 mM sodium phosphate and 150 mM NaCl, pH 7.4) containing 1% NP-40 and protease inhibitor cocktail (Sigma-Aldrich)]. For the proteomic analysis of the XC24 antigen, HepG2 cell lysates prepared with NP40 lysis buffer were immunoprecipitated with XC24 antibody-conjugated beads. Antibody-conjugated beads were prepared using immunoprecipitation kit (Thermo). The precipitated beads were resolved by 10% SDS-PAGE and analyzed by western blotting or Coomassie Blue staining. XC24 reactive antigen bands were then excised from SDS-PAGE gel and in gel-digested with trypsin (Trypsin Gold, Mass Spectrometry Grade; Promega, Fitchburg, Wisconsin, USA). The peptide extracts of in-gel digestion were analyzed by nano-liquid chromatography electrospray ionization–tandem mass spectrometry (nano-LC–ESI–MS/MS), as previously described [28].

To confirm the result of mass spectrometric analysis, HepG2 cells were transfected with AccuTarget™ siRNA against human SF3B1 or HYOU1 (Bioneer Corporation, Daejeon, Korea; si-SF3B1: sense 5′-CGA AGA UCG CCA AGA CUC A(dTdT)-3′, antisense 5′-UGA GUC UUG GCG AUC UUC G(dTdT)-3′; si-HYOU1: sense 5′-CAG AGA UGG ACC AGA UCU U(dTdT)-3′, antisense 5′- AAG AUC UGG UCC AUC UCU G(dTdT)-3′) using Lipofectamine RNAiMAX reagent (Thermo), and then reverse transcription-polymerase chain reaction (RT-PCR) and western blot analysis were performed 72 h after transfection. AccuTarget™ Negative Contol siRNA (Bioneer) was used as negative control. Total RNA was extracted from cells using Qiagen RNA extraction kit (Qiagen, Hilden, Germany) and the first-strand cDNA was synthesized using Superscript III (Thermo). RT-PCR was performed using the following primer pairs (Bioneer; SF3B1: forward 5′-CTC GAG ATG GCT GCA TTG CGT CAG ATT AC-3′, reverse 5′-CTC GAG CAT TAC TGA GTC CTC TGT AGT-3′; GAPDH forward 5′-AGA GAC TGG AGC CAT TAC TTC-3′, reverse 5′-CAA CCT CAG CAG ACT GTG TG-3′). To further confirm whether XC24 antigen is SF3B1, the immunoprecipitated complex with anti-SF3B1 antibody (Bethyl Laboratories, Montgomery, TX, USA) was probed with XC24 antibody or vice versa.

For the immunofluorescence microscopy analysis, cells plated on 18 × 18-mm glass coverslips in 6-well plates were treated with BD cytofix/cytoperm solution to fix and permeabilize the cells. Then, cells were incubated with antibodies (XC24 or anti-SF3B1 antibody) diluted in BD cytoperm/wash solution (5 μg/mL) and with FITC-conjugated secondary reagents. The immuno-stained coverslips were mounted with Vectashield medium containing DAPI (Vector Laboratories, Burlingame, CA, USA) and analyzed using a Zeiss LSM510 Meta microscope (Carl Zeiss MicroImaging, Jena, Germany).

Subcellular localization of SF3B1 or XC24 antigen was determined by western blotting on subcellular fractionated cell lysates. Cells were washed twice with PBS and lysed using NE-PER nuclear and cytoplasmic extraction reagents (Thermo). Membranes were probed with one of the following antibodies: anti-Lamin B1 (nuclear marker; Santa Cruz Biotechnology Inc. Santa Cruz, CA, USA), anti-GAPDH (cytoplasm marker, Santa Cruz), and anti-SF3B1 antibody or XC24 monoclonal autoantibody.

To evaluate the expression of SF3B1 in HCC tissues, cell lysates prepared using RIPA buffer as previously described [28] or tissue lysates prepared by homogenization and sonication were analyzed by western blot analysis. Exosomes collected from the cell culture media by ultracentrifugation [30] was resolved in RIPA buffer and analyzed by western blotting. The protein concentration was determined by the Bradford method (Bio-Rad, Hercules, CA, USA), and equal amounts of protein (50 μg) were resolved by SDS-PAGE and transferred onto a PVDF membrane (Millipore, MA, USA). The membranes were probed with XC24 monoclonal antibody or anti-SF3B1 antibody; β-actin was probed as a loading control (Abcam) and an exosome marker ALIX was detected as with anti-ALIX antibody (Millipore). Calexin, an endoplasmic reticulum marker, was probed (Abcam) as cell contamination marker. Positive bands were detected by horseradish peroxidase (HRP)-linked anti-mouse IgG/M/A antibody (Abcam) or other corresponding secondary reagents, followed by enhanced chemiluminescence reagents (GE Healthcare Life Sciences, Pittsburgh, PA, USA). Band intensity was quantified using Image J (NIH, USA) and the relative intensity compared to β-actin was calculated.

For the staining of mouse tissue, 4-μm-thick sections were cut from the formaldehyde-fixed and paraffin-embedded tissue specimens and mounted on charged glass slides as described previously [15]. Tissue section samples were incubated overnight at 4 °C with anti-SF3B1 antibody. The sections were then incubated with HRP-conjugated secondary antibody for 30 min at room temperature, and 3,3′-diaminobenzidine (DAB) substrate chromogen solution was applied. Finally, the sections were counterstained with hematoxylin, dehydrated using graded alcohol and xylene series, and mounted with Permount (VWR International, Strasbourg, France). The photomicrographs were acquired at 200× or 400× magnification. DAB intensity was quantified using Image J (NIH) and plotted.

### Evaluation of anti-SF3B1 autoantibody in human patients with hepatocellular carcinoma

We screened the conformational epitopes against XC24 antibody with the cyclic peptide library Ph.D.™-C7C [New England Biolabs (NEB), Ipswich, MA, USA] to use them as detection antigens of SF3B1 autoantibody instead of using a recombinant SF3B1 protein. Screening of conformational epitopes was already proven to be successful in the study of anti-FASN and anti-CK8/18 autoantibody biomarkers [28, 31]; Panning was repeated four times, and sequencing of selected mimotope phages was performed following the manufacturer’s instructions. To confirm the specificity of selected epitopes displayed on M13 phages, phage ELISA was performed [28]. Briefly, the ELISA plate (MaxiSorp™; Thermo) was coated with a selected phage (10^10^ pfu/well) in 0.1 M sodium carbonate buffer (pH 8.6). After the wells were blocked with protein-free blocking buffer (Thermo), primary antibody solution (100 ng purified XC24 or XC20 monoclonal antibody in 100 μl blocking buffer) was added and incubated at room temperature for 2 h. HRP-linked anti-mouse IgG/M/A antibody (Sigma-Adrich; 1:2000 diluted in blocking buffer) was used as a secondary reagent. Tetramethylbenzidine (TMB)-based substrate solution (Thermo) was used for color development. When evaluating the effect of cyclic conformation on peptide antigenicity, cyclic phages were reduced and alkylated and then coated onto the ELISA plates described previously [28]. For the competitive FACS analysis to confirm the specificity of selected epitopes, the primary antibody XC24 or XC20 was pre-incubated with the indicated epitope-displaying phages (indicated pfu/100 μl reaction) and used for cell staining. Competitive western blotting was also performed. The HepG2 or other cell lysates were resolved by SDS-PAGE and western blotted with primary antibody XC24 or XC20, which was pre-incubated with epitope-displaying phages (10^11^ pfu of indicated phages/100 μl reaction).

For the preparation of epitope-display coating antigens for human serum ELISA, we constructed XC24p11 epitope-fused streptavidin expression vector. The streptavidin-coding region was PCR-amplified from *Streptomyces avidinii* (ATCC 27419) and cloned into a *Not*I/*Xho*I-digested pET28a (+) vector. The DNA coding the cyclic peptide XC24p11 epitope sequence (-CDATPPRLC-) with restriction enzyme sites *Nde*I and *Not*I was synthetized (Bioneer; foward 5′-tatg ggt ggt gcg TGC GAC GCG ACC CCG CCG CGT CTG TGC ggt gga ggt tcg gcc-3′, reverse 5′-ggc cgc cga acc tcc acc GCA CAG ACG CGG CGG GGT CGC GTC GCA cgc acc acc ca-3′; underlined sequences correspond to XC24p11 epitope sequence) and cloned into the streptavidin-cloned pET28a (+) vector. The cyclic epitope gene was separated from the N-terminal His-tag and streptavidin gene by a linker encoding the amino acids GSGSA. DsbA, a bacterial thiol disulfide oxidoreductase (TDOR), was also cloned into the cyclic epitope-streptavidin cloned-pET28a (+) vector to catalyze the intrachain disulfide formation of the cyclic epitope. In addition, for the effective disulfide bond formation, SHuffle^®^ T7 (NEB) was used as a host cell, which is an *E. coli* K12 strain suitable for T7 protein expression with enhanced capacity to correctly fold proteins with multiple disulfide bonds in the cytoplasm.

Transformants of *E. coli* strain SHuffle^®^ T7 with XC24p11 epitope-fused streptavidin expression vector were grown overnight at 30 °C in 2× YT broth containing kanamycin (50 μg/ml). The culture was diluted 100-fold into fresh medium and grown in a shaking incubator at 30 °C. When the culture attained an absorbance at 600 nm of 4, isopropyl-β-d-thio-galactopyranoside (Sigma-Aldrich) was added to a final concentration of 1 mM, and incubation was continued overnight at 25 °C. Cells were harvested at 24–26 h post-inoculation by centrifugation, washed with TBS [10 mM Tris–HCl and 150 mM NaCl (pH 7.4)], and pelleted by centrifugation. The cells were resuspended in an ice-cold solution of 5% glycerol, 50 mM Tris–HCl (pH 7.4), and 2.0 mg/ml lysozyme, placed on ice for 30 min, and the cell suspension was sonicated to obtain the cell lysate. The lysate solution was centrifuged (10,000×*g* for 90 min, 4 °C) to pellet the cellular debris. The supernatant was decanted and filtered through a 0.2-μm filter. The lysate solution, to which NaCl and imidazole were added to a final concentration of 500 and 10 mM, respectively, was then applied over a Talon affinity column (Takara Bio, Mountain View, CA, USA) equilibrated in 50 mM Tris–HCl (pH 7.4) containing 0.25 M NaCl, 10 mM imidazole, and 5% glycerol. The column was washed with 10 column volumes of equilibration buffer and then eluted with an imidazole gradient up to 0.5 M in equilibrium buffer. The eluate fractions containing streptavidin antigen were pooled and concentrated using Amicon^®^ Ultra Centrifugal Filters (Merck Millipore, Darmstadt, Germany) down to 1 ml. The concentrate was applied to a HiLoad 16/600 Superdex 200 prep grade column (GE) equilibrated with PBS containing 1 mM β-mercaptoethanol. Each fraction obtained by size-exclusion chromatography was analyzed by SDS-PAGE and western blotting, and fractions containing tetrameric epitope-fused streptavidin were pooled and used for further analysis and human serum ELISA.

The ELISA plate, MaxiSorp-, or biotin-coated plates (Thermo) were coated with the indicated amounts of epitope-fused streptavidin in 0.1 M sodium carbonate buffer (pH 8.6) overnight at 4 °C. After the wells were blocked with protein-free blocking buffer, XC24 primary antibody solution (containing the indicated amount of purified monoclonal antibody in 100 μl blocking buffer) was added and incubated at room temperature for 2 h. HRP-linked anti-mouse IgG/M/A antibody (Sigma-Aldrich; 1:2000 diluted in blocking buffer) was used as a secondary reagent. TMB solution was used for color development. For the detection of reactivity of patient sera to XC24p11-streptavidin, the MaxiSorp plates were coated with XC24p11 epitope-fused streptavidin at 500 ng/well, and after blocking with protein-free blocking buffer as described above, the plates were treated with albumin-depleted human sera (1:1000 diluted in blocking buffer) and detected by HRP-conjugated anti-human IgG/M/A antibody (1:2000 diluted in blocking buffer). Albumin depletion of the human serum was performed using Affi-Gel^®^ Blue Gel following the manufacturer’s instructions (Bio-Rad). Empty-streptavidin (Eph) without a peptide epitope insert was used as control coating antigen. Alpha-fetoprotein (AFP) levels in human sera were evaluated with Human alpha-Fetoprotein Quantikine ELISA Kit (R&D systems, Minneapolis, MN, USA).

### Statistical analysis

Data are presented as the mean ± SD. The two-tailed Student’s t-test was used to evaluate significance; p values < 0.05 were considered statistically significant. The sensitivity and specificity of anti-XC24p11 autoantibody or AFP for the diagnosis of HCC was evaluated using receiver-operating characteristics (ROC), leading the estimates of the area under the curve (AUC), with 95% confidence intervals. Statistical analysis was carried out using Prism 7 software (GraphPad Software, La Jolla, CA, USA). *ns* p > 0.05, * p ≤ 0.05, ** p ≤ 0.01, *** p ≤ 0.001, **** p ≤ 0.0001.

## Results

### TA autoantibody against SF3B1 was identified in HBx-Tg HCC model mouse

**Fig. 1:**
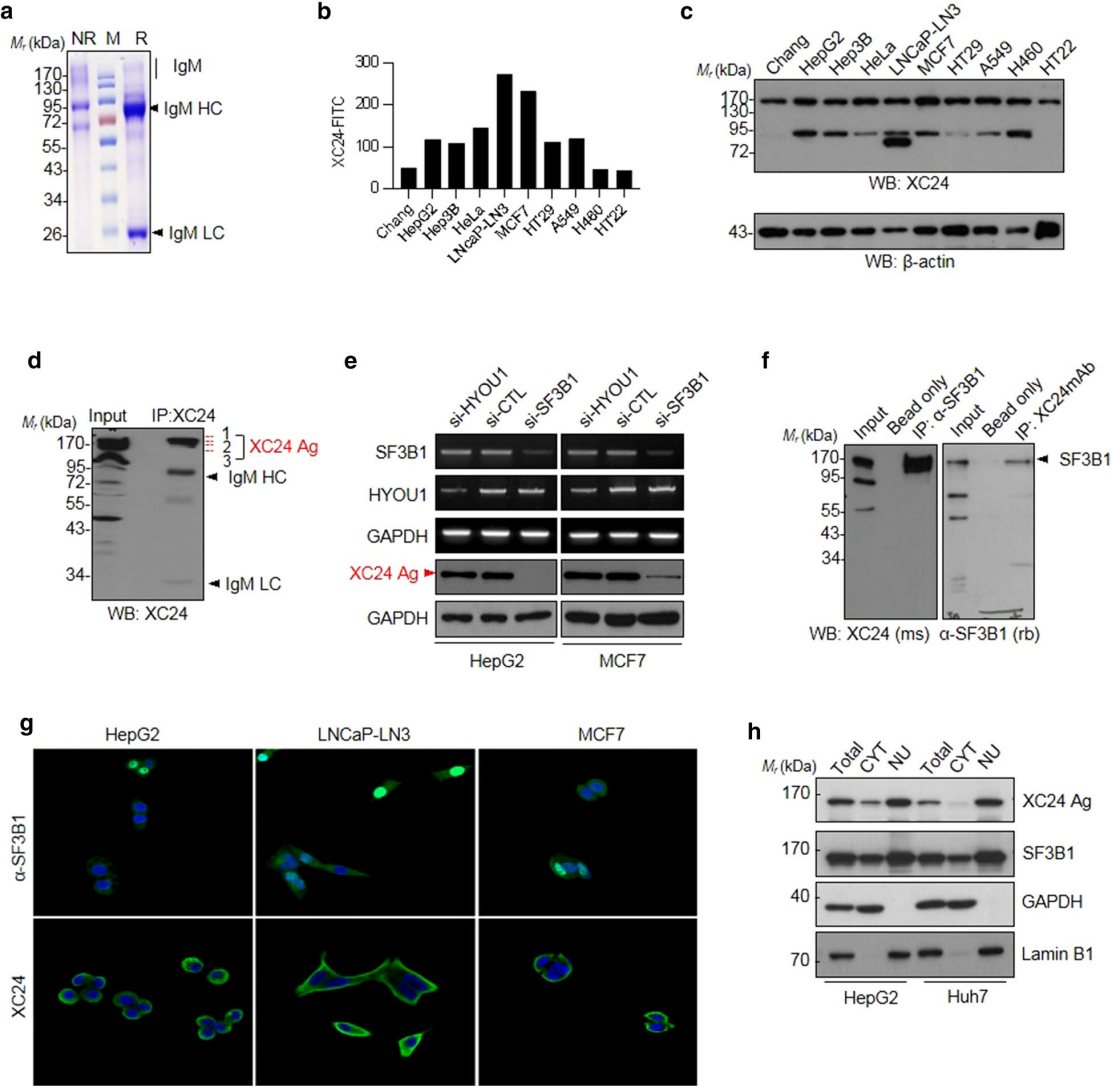
Anti-SF3B1 autoantibody, XC24, was identified in HBx-transgenic HCC model mouse. **a** Purified XC24 monoclonal autoantibody analyzed by SDS-PAGE (NR, non-reduced; R, reduced sample; M, molecular weight marker); **b** FACS analysis of intracellular-stained tumor cell lines with XC24 (immortalized liver cell Chang cell, HepG2 and Hep3B hepatocellular carcinoma cells as well as non-hepatocellular carcinoma cells, including cervical (HeLa), prostate (LNCaP-LN3), breast (MCF7), colon (HT29), lung (A549 and H460) cancer cells and mouse neuronal cell (HT22); **c** Western blot analysis of specific antigen against XC24 TA autoantibody; **d** Protein identification by mass spectroscopic analysis. Tryptic digest peptides derived from proteins in three bands indicated as XC24 Ag were extracted and analyzed by mass spectrometry (Table 1); **e** Verification of XC24 antigen as SF3B1 by knockdown assay using siRNA. The transcripts of *SF3B1* and *HYOU1* in knockdown cells were examined by RT-PCR and the protein level of XC24 antigen was confirmed by western blot analysis; **f** Verification of XC24 autoantibody-specific antigen as SF3B1 by immunoprecipitation and western blot analysis. SF3B1 immunoprecipitated with a commercial rabbit anti-SF3B1 antibody was analyzed by western blotting with XC24 mouse monoclonal antibody or vice versa (Additional file 2: Fig. S1); **g** Immunofluorescence intracellular staining of tumor cells with anti-SF3B1 antibody or XC24 monoclonal antibody; **h** Western blot analysis of subcellular localization of XC24 antigen (Additional file 3: Fig. S2). GAPDH was used as a cytoplasmic marker and Lamin B1 was used as a nuclear marker

To identify the TA autoantibody biomarkers, each autoantibody produced by HBx-Tg mice B cell hybridoma clones was purified and reactivity to human cancer cells were analyzed. XC24, one of such TA autoantibodies (Fig. 1a), showed specific responses to HepG2 and other cancer (Hep3B, HeLa, LNCaP-LN3, MCF7, HT29, A549, H460) or non-cancer cell lines (Chang, HT22) (Fig. 1b). XC24 also could be applied to western blot analysis, which showed its target antigen is a protein with molecular weight of about 170 kDa (Fig. 1c). To identify an XC24 antibody-specific antigen, HepG2 cell lysates were immunoprecipitated with XC24-conjugated beads and resolved by SDS-PAGE. The protein bands corresponding to XC24 antigen, which was confirmed by western blotting, was then excised and in-gel digested with trypsin (Fig. 1d). The peptide extracts of in-gel digestion were analyzed by nano-liquid chromatography electrospray ionization-tandem mass spectrometry (nano-LC–ESI–MS/MS). Several protein candidates were identified, including splicing factor 3b subunit 1 (SF3B1) and hypoxia up-regulated protein 1 (HYOU1) (Table 1), among which the highest Mascot Score was shown by SF3B1. To confirm the result of mass spectrometric analysis, tumor cells were transfected with small interfering RNAs (siRNA) against SF3B1 or HYOU1, and total cell lysates were analyzed by western blotting. As shown in Fig. 1e, the protein bands stained with XC24 autoantibody in HepG2 or MCF7 cell lysate was disappeared when SF3B1 was knocked down by siRNA. The immunoprecipitated complex with anti-SF3B1 antibody was also probed with XC24 antibody or vice versa to confirm that XC24 antigen is SF3B1 (Fig. 1f, Additional file 2: Fig. S1). SF3B1 is a component of the pre-mRNA splicing multi-protein complex SF3B, which functions mainly in nucleus [32]. To confirm the XC24 autoantibody-specific antigen as SF3B1, the intracellular staining with XC24 antibody was compared with anti-SF3B1 antibody staining. As shown in Fig. 1g, commercial antibody staining against SF3B1 showed nuclear as well as cytoplasmic staining. XC24 autoantibody staining was also localized in nucleus as well as cytoplasm, but mainly in cytoplasm and plasma membrane. Occasionally, intracellular cell staining with antibodies may be influenced by its accessibility to subcellular compartments. To confirm again XC24 antigen as SF3B1, cellular fractionation and western blot analysis was performed. When nuclear envelope was disrupted XC24 antibody also detect nuclear fraction more than cytoplasmic fraction as commercial anti-SF3B1 antibody (Fig. 1h, Additional file 3: Fig. S2). Collectively, XC24 antibody was revealed as a tumor-associated antibody against SF3B1.

**Table 1:** Mass spectrometric analysis of XC24 antigen

Band no.	Gene symbol	Accession no.	Predicted MW (Da)	No. of peptide matches	Score
1	SF3B1	IPI00026089	145,738	54	2053
	SMARCC1	IPI00234252	122,790	16	631
	PRSS3	IPI00015614	32,508	1	91
	CCAR1	IPI00217357	132,739	1	59
	EEF1A2	IPI00014424	50,438	1	53
2	U2SURP	IPI00143753	118,219	11	805
	SF3B2	IPI00221106	100,165	10	511
	HYOU1	IPI00000877	111,266	7	216
	SF3B1	IPI00026089	145,738	5	200
	LRPPRC	IPI00783271	157,805	4	164
3	LRPPRC	IPI00783271	157,805	7	486
	SF3B2	IPI00221106	100,165	6	323
	SF3B3	IPI00300371	135,492	6	258
	SF3B1	IPI00026089	145,738	6	202
	U2SURP	IPI00790699	72,479	2	182

SF3B1, splicing factor 3B subunit 1; SF3B2, splicing factor 3B subunit 2; SF3B3, splicing factor 3B subunit 3; HYOU1, hypoxia up-regulated protein 1; U2SURP, isoform 1 of U2 snRNP-associated SURP motif-containing protein; LRPPRC, leucine-rich PPR motif-containing protein, mitochondrial; SMARCC1, SWI/SNF complex subunit SMARCC1; PRSS3, protease, serine 3; CCAR1, isoform 1 of cell division cycle and apoptosis regulator protein 1; EEF1A2, elongation factor 1-alpha 2

**Fig. 2:**
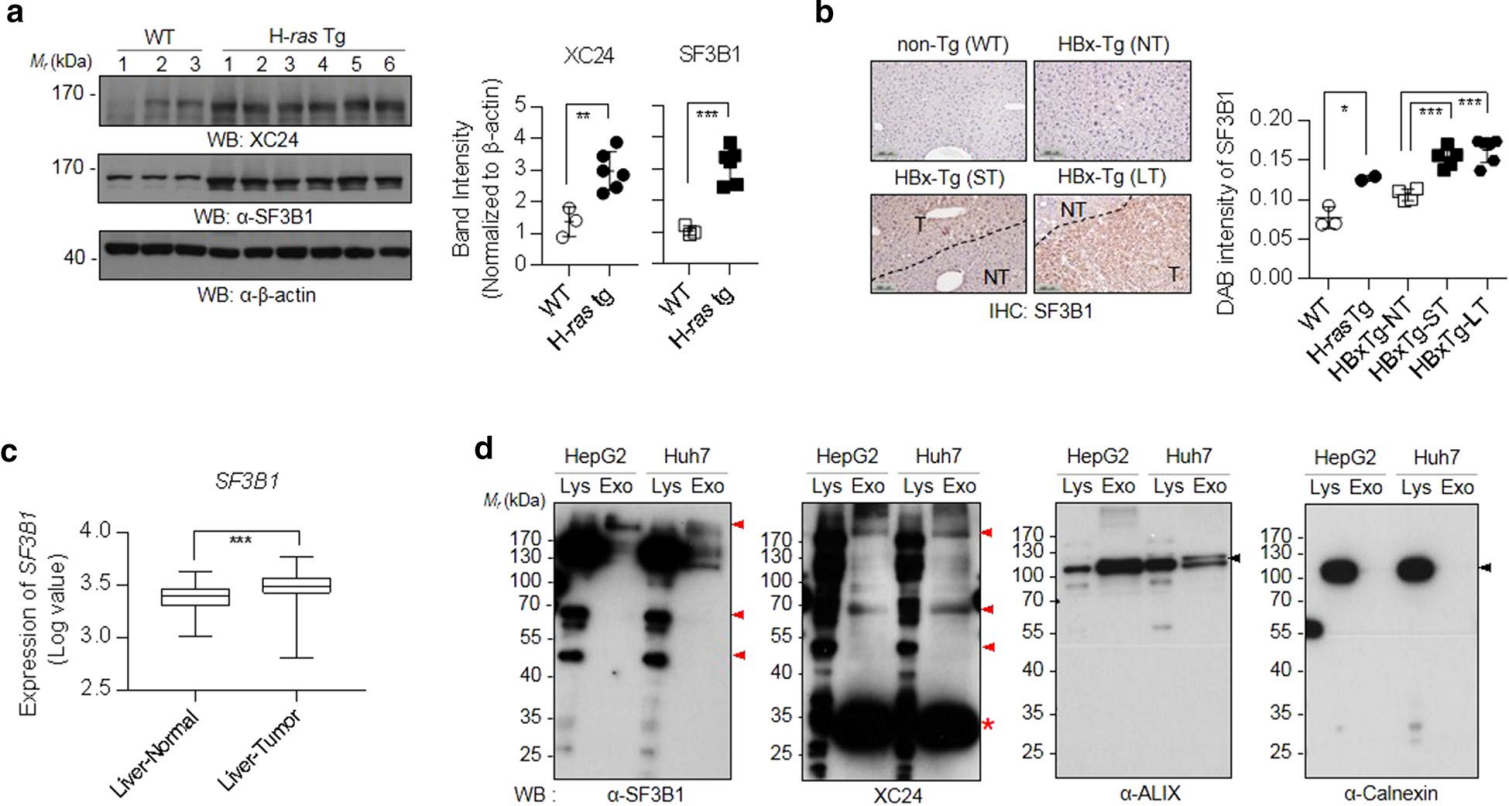
SF3B1 is elevated in HCC tissues of HCC-model mice and human HCC patients. **a** Western blot analysis of SF3B1 expression in H-*ras*12V transgenic HCC model mice. Liver tissues of three normal mice and six H-*ras*12V-Tg mice were homogenized, lysed in RIPA buffer, and resolved by 10% SDS-PAGE/western blotting. Band intensities on the western blot were quantified by Image J and normalized to that of β-actin; **b** Representative immunohistochemical staining with anti-SF3B1 antibody in liver tissues of HCC model transgenic mice (HBx-Tg and H-*ras*12V-Tg; T, tumor; NT, non-tumor region; ST, small tumor; LT, large tumor). DAB intensities of IHC staining (Additional file 4: Fig. S3) were quantified by Image J and plotted; **c** Analysis of SF3B1 mRNA expression in human normal liver and liver cancer using GENT database (http://medicalgenome.kribb.re.kr/GENT/); **d** Western blot analysis of SF3B1 in exosomes from HepG2 or Huh7 cell cultured media using anti-SF3B1 antibody or XC24 autoantibody. As subcellular fraction markers, ALIX (exosome marker) and calnexin (ER marker to examine cell contamination) were also probed. The amount of protein loaded in each lane is 5ug. Lys: cell lysate, Exo: exosome lysate

Northern blot analysis and whole mount in situ hybridization revealed SF3B1 to be ubiquitously expressed in a variety of adult tissues and mid-gestation embryos [32]. Recently, SF3B1 mutations have been described in several malignancies including HCC [33–36], which cause aberrantly spliced transcripts, thus providing a link between SF3B1 mutations and disease [37]. Considering its ubiquitous expression in human tissues, SF3B1 must be recognized as self in the healthy human body; however, a certain alteration in the antigenic presentation of SF3B1, such as its expression level, localization, mutation or their combinations, can induce a specific autoantibody during tumorigenesis. We examined first, therefore, the SF3B1 expression in liver tissues. Western blot analysis showed that SF3B1 expression was significantly increased by about 2- or 3-fold (p < 0.05) in HCC tissues of H-*ras*12V transgenic (H*ras*12V-Tg) mice, another mouse model of liver cancer [29], compared to normal liver tissues (Fig. 2a). The elevation of SF3B1 was further confirmed by immunohistochemistry analysis. In the tumor region of the liver tissue of HBx-Tg mice (especially in large tumor region), SF3B1 was increased compared to that of the non-tumor region (Fig. 2b, Additional file 4: Fig. S3). Public gene expression database analysis using GENT analysis (Gene Expression across Normal and Tumor tissue; http://medicalgenome.kribb.re.kr/GENT/) also showed the elevation of *SF3B1* in human liver tumor tissues compared to normal tissues (Fig. 2c). Collectively, SF3B1 expression is increased in liver cancer, which may be the primary cause of induction of abnormal autoimmune-response.

For the induction of TA autoantibodies, the elevated intracellular antigen should be released to the extracellular region, in which antigen can stimulate immune system. Cell death or necrosis accompanied by tumorigenesis has been assumed to be a predominant condition that exposes intracellular components to extracellular space [38]. Recent studies on TA exosomes, however, showed that intracellular antigens can be exposed to the extracellular region even without cancer cell necrosis. SF3B1 had already been confirmed as a component of TA exosomes derived from various cancers such as colorectal and ovarian cancer [39, 40]. Therefore, we examined whether SF3B1 is secreted as an exosomal component from HCC by analyzing exosomes derived from HCC cancer cell lines. Exosomes from the HepG2 or Huh7 cancer cells were collected by ultracentrifugation and probed with XC24 as well as with commercial anti-SF3B1 antibody (Fig. 2d). Antigen bands stained with XC24 or anti-SF3B1 antibody are overlapped at the same protein band of molecular weight of about 170 kDa. Several protein bands less than molecular weight of 170 kDa were also detected with anti-SF3B1 or XC24 antibody at the same locations (Fig. 2d; indicated by red arrow head), which may be the degradation fragments of SF3B1. Interestingly, an excess amount of low molecular weight protein (about 30 kDa; indicated by asterisk) was detected by XC24 autoantibody, which also may be a fragment derived from SF3B1, but not detectable with commercial anti-SF3B1 antibody.

### Human serum ELISA detecting SF3B1 autoantibody with XC24p11 epitope can be used for the diagnosis of HCC

**Fig. 3:**
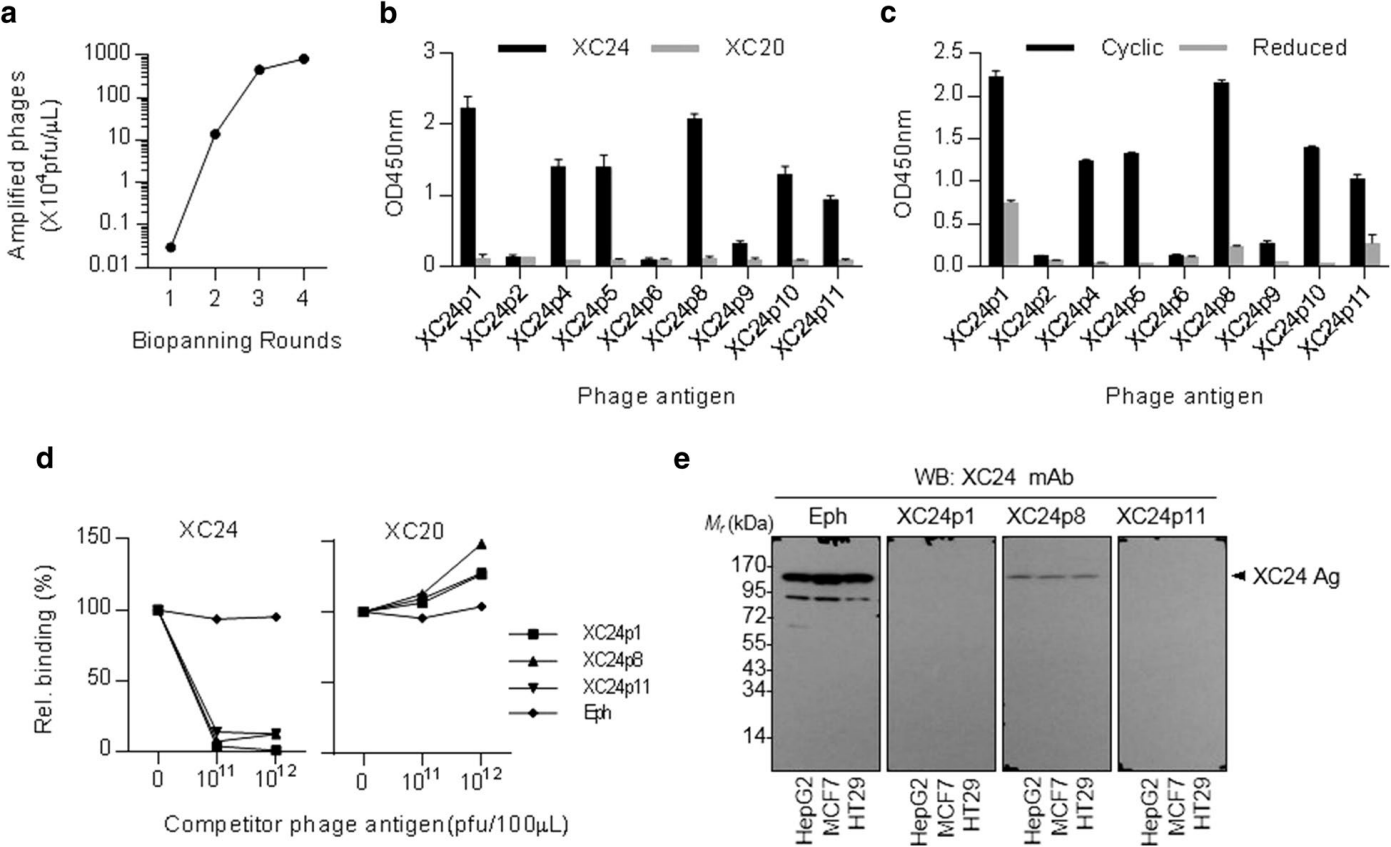
Specific peptide epitopes against XC24 antibody screened from phage display random cyclic hepta-peptide library. **a** Biopanning with XC24 autoantibody against M13 phage display random cyclic hepta-peptide library to screen mimotopes specific to XC24 autoantibody; **b** Phage ELISA of selected epitopes against XC24 autoantibody. XC20, another HBx-Tg mouse-derived autoantibody, was used as a control antibody; **c** Effect of reducing disulfide bonds on the reactivity of cyclic peptide mimotopes against XC24 autoantibody. The cyclic mimotope display phages, either untreated (Cyclic) or treated with dithiothreitol and iodoacetamide (Reduced), were tested for their responses to XC24 autoantibody; **d** Competitive inhibition of XC24 autoantibody binding to HepG2 cells by XC24 mimotopes (XC24p1, XC24p8 and XC24p11). Fixed and permeabilized HepG2 cells were treated with XC24 autoantibody, which was pre-adsorbed with the indicated phages (10^11^ or 10^12^ pfu). The reactivity of the phage-added reaction was compared to that of phage-free reaction; **e** Western blot analysis of competitive inhibition of XC24 autoantibody binding to tumor cells by XC24 mimotopes (XC24p1, XC24p8, and XC24p11). Cell lysates were blotted and treated with XC24 autoantibody, which was pre-adsorbed with XC24 mimotope display M13 phages (10^11^ pfu) or with control M13 phage (Eph)

**Fig. 4:**
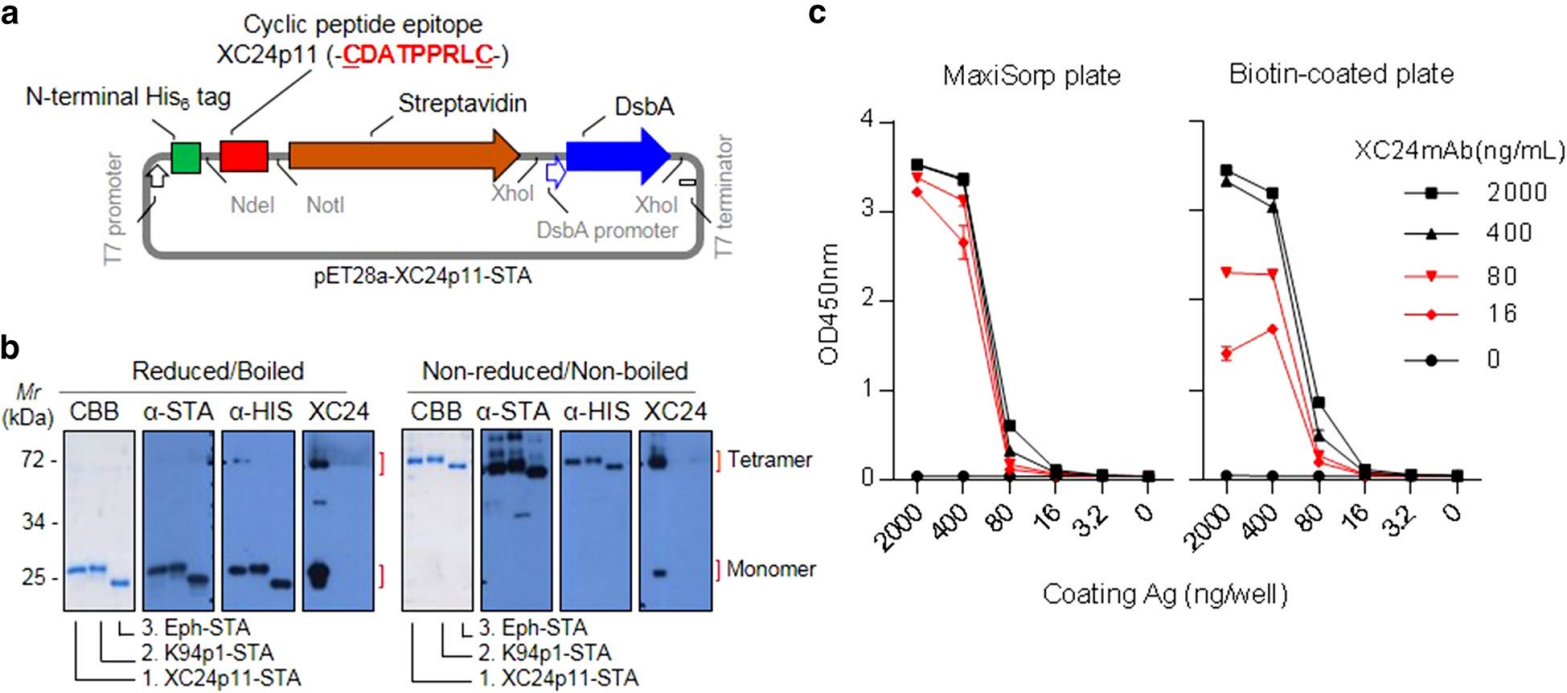
XC24p11-streptavidin mimicked effectively the antigenic structure on SF3B1 for the binding of XC24 autoantibody. **a** Construction of XC24p11 cyclic peptide-fused streptavidin (XC24p11-STA) expression vector. Dsb A, a bacterial thiol disulfide oxidoreductase, was co-expressed to enhance disulfide bond formation of the XC24p11 cyclic epitope; **b** SDS-PAGE and western blot analysis of XC24p11-STA (5 or 1 μg/each lane). To confirm the monomeric form of XC24p11-STA, purified XC24p11-STA was mixed with reducing SDS-PAGE sample buffer, boiled, and analyzed by 10% SDS-PAGE and western blotting. Tetrameric XC24p11-STA was also confirmed by the analysis of non-reduced and non-boiled antigen in SDS-PAGE sample buffer on 10% SDS-PAGE and western blotting (CBB, Coomassie Brilliant Blue staining; α-STA, anti-streptavidin antibody staining; α-HIS, anti-His_6_ antibody staining; XC24, XC24 autoantibody staining). K94p1-STA, another autoantigenic epitope-fused STA (K94p1 epitope, -CISPDAHSC-, previously reported [31]) and epitope-free STA (Eph-STA) were also analyzed as controls; **c** XC24p11-STA ELISA on MaxiSorp- or biotin-coated plates. The conditions of ELISA are described in detail in Materials and Methods

The results described above implicates that SF3B1 is increased in HCC and can induce a specific autoantibody as it is secreted from cancer cells as an exosomal component. Since the characteristics of human HCC cells are similar to those of HCC model mouse [15–27], human SF3B1-specific autoantibody is also expected to be induced in human HCC patients. Thus, we had screened the conformational epitopes of XC24 antibody from a cyclic peptide library to use them as detection antigen of anti-SF3B1 autoantibody. Screening conformational epitopes using a cyclic peptide display phage library was already proven to be successful in our previous studies [28, 31]; hence, the same screening method was applied for anti-SF3B1 autoantibody. Bio-panning of M13 phage library displaying 10^11^ to 10^13^ cyclic peptides with XC24 autoantibody was repeated four times and specific binding phages were amplified about 1000 times (Fig. 3a). The epitope sequences of amplified phages from about 20 phage plaques were determined and 10 different epitopes were obtained (Table 2). In the ELISA using these cyclic peptide epitope-displaying M13 phages as the coating antigens, XC24 antibody showed different titers for each phage display epitope, and the reactivity against XC24p1, XC24p4, XC24p5, XC24p8, XC24p10, and XC24p11 epitopes was shown to be high (Fig. 3b). These epitopes did not show any reactivity against another autoantibody, XC20, indicating that the selected cyclic peptides display XC24 antibody-specific epitopes. All of selected epitope sequences showed a consensus sequence motif of xDxTPxx type (D: Aspartic acid; T: Threonine; P: Proline; x: diverse amino acids except cysteine), although this sequence motif of conformational epitope was not found in the protein primary sequence of SF3B1. To confirm whether the specificity of cyclic peptide epitopes against XC24 antibody depends on their cyclic structures, the antigenic cyclic peptide-displaying phages were reduced with dithiothreitol and alkylated, and were then subjected to ELISA. As shown in Fig. 3c, the reactivity of antigenic phages to the XC24 antibody disappeared when the disulfide bonds were reduced and the cyclic structures were linearized, confirming that the cyclic form of these epitopes is important for their binding to XC24 autoantibody. Competitive FACS analysis was performed to confirm how well the antigenic epitopes simulate the actual intracellular antigenic structures. After HepG2 cells were fixed and permeabilized, maintaining most of the structural features of antigenic determinants, they were probed with XC24 or XC20 antibodies and specific bindings of each antibody were detected (Fig. 3d). However, by adding antigenic epitope-displaying phages, such as XC24p1, XC24p8, or XC24p11, the XC24 antibody-specific binding was inhibited to almost zero. These phages did not affect the XC20 antibody binding to HepG2 cells; rather, they enhanced the antibody binding, maybe by increasing non-specific reactions. The antigenic specificity of these epitope-displaying phages was also confirmed by competitive western blot analysis (Fig. 3e). The XC24 antibody response to the HepG2, MCF7 or HT29 cell lysate on western blot was completely inhibited by XC24-specific epitope phages (XC24p1, XC24p8, and XC24p11), but not by epitope-free M13 phage (Eph). Collectively, we confirmed that the cyclic peptide epitopes including XC24p11, which sufficiently mimics the conformational characteristics of the XC24 autoantibody epitope, can be used as a detection antigen of XC24 autoantibody instead of cellular SF3B1 with a molecular weight of 155 kDa. In order to establish practical diagnostic tests, it is necessary to mass-produce the coated antigens. For this purpose, we fused the cyclic peptide epitope sequences to the amino-terminus of matured streptavidin (without the secretion leader sequence) and expressed as a streptavidin tetramer in *E. coli* (Fig. 4a). Among X24-reactive epitopes, XC24p11 was expressed on streptavidin carrier, because of protein expression efficiency. DsbA, a bacterial thiol disulfide oxidoreductase (TDOR), was also cloned into an antigen-expressing pET28a vector and expressed simultaneously with cyclic peptide display streptavidin to induce the correct disulfide bond necessary for formation of the cyclic peptide structure. In addition, SHuffle^®^ T7 was used as a host cell for effective disulfide bond formation. The expressed epitope-displaying streptavidins were purified using His-tag affinity chromatography and Superdex 200 size-exclusion chromatography. The streptavidin tetramer was confirmed by its size of about 80 kDa in the elution profile of Superdex 200 size-exclusion chromatography and SDS-PAGE (Fig. 4b). For the detection of human serum autoantibody, the conditions of each step in ELISA were optimized. The antibody binding to streptavidin antigens was compared on MaxiSorp plate and biotin-coated plate to select a solid phase suitable for coating epitope-fused streptavidin antigens (Fig. 4c). XC24 antibody binding to the XC24p11-streptavidin antigen was saturated at about 400 ng/well in both type of coating plates. However, antibody binding was diminished when the amount of coating antigen in the biotin-coated plate was increased. The decrease of antibody binding was particularly noticeable when the amount of primary antibody was low. When considering these properties of the streptavidin antigen on coating plates and the sensitivity of ELISA, the MaxiSorp plate seems to be more suitable for the human serum autoantibody ELISA, which must detect the autoantibody more stably even at low concentrations. Protein-free blocking buffer was used for plate blocking and human serum was pretreated with albumin depletion resin to remove albumin, which is a typical source of reducing potential in blood, and diluted 50-fold in protein-free blocking buffer.

**Table 2:** Epitope Peptide Sequences selected against XC24

Phage no.	Amino Acid Sequences (-C-X_1_X_2_X_3_X_4_X_5_X_6_X_7_-C-)^*^							Frequency^**^
	X_1_	X_2_	X_3_	X_4_	X_5_	X_6_	X_7_	
XC24p1	T	*D*	M	*T*	*P*	G	A^***^	1/11
XC24p2	*D*	R	*T*	*P*	Y	R	V	1/11
XC24p3	F	*D*	A	*T*	*P*	H	V	1/11
XC24p4	T	*D*	G	*T*	*P*	H	A	1/11
XC24p5	T	*D*	Q	*T*	*P*	Y	V	2/11
XC24p6	*D*	R	*T*	*P*	G	R	P	1/11
XC24p8	T	*D*	K	*T*	*P*	G	F	1/11
XC24p9	S	*D*	R	*T*	*P*	Y	M	1/11
XC24p10	T	*D*	R	*T*	*P*	W	A	1/11
XC24p11	*D*	A	*T*	*P*	P	R	L	1/11

^*^The randomized amino acid sequence (X_1_–X_7_) is flanked by a pair of cysteine residues (C). Under nonreducing conditions the cysteines will spontaneously form a disulfide cross-link, resulting in phage display of cyclized peptides

^**^ The epitope sequences of amplified phages from about 20 phage plaques were determined and 10 different epitopes were obtained

^***^Consensus peptide sequence motif (x*D*x*TP*xx) is shown in italics

**Fig. 5:**
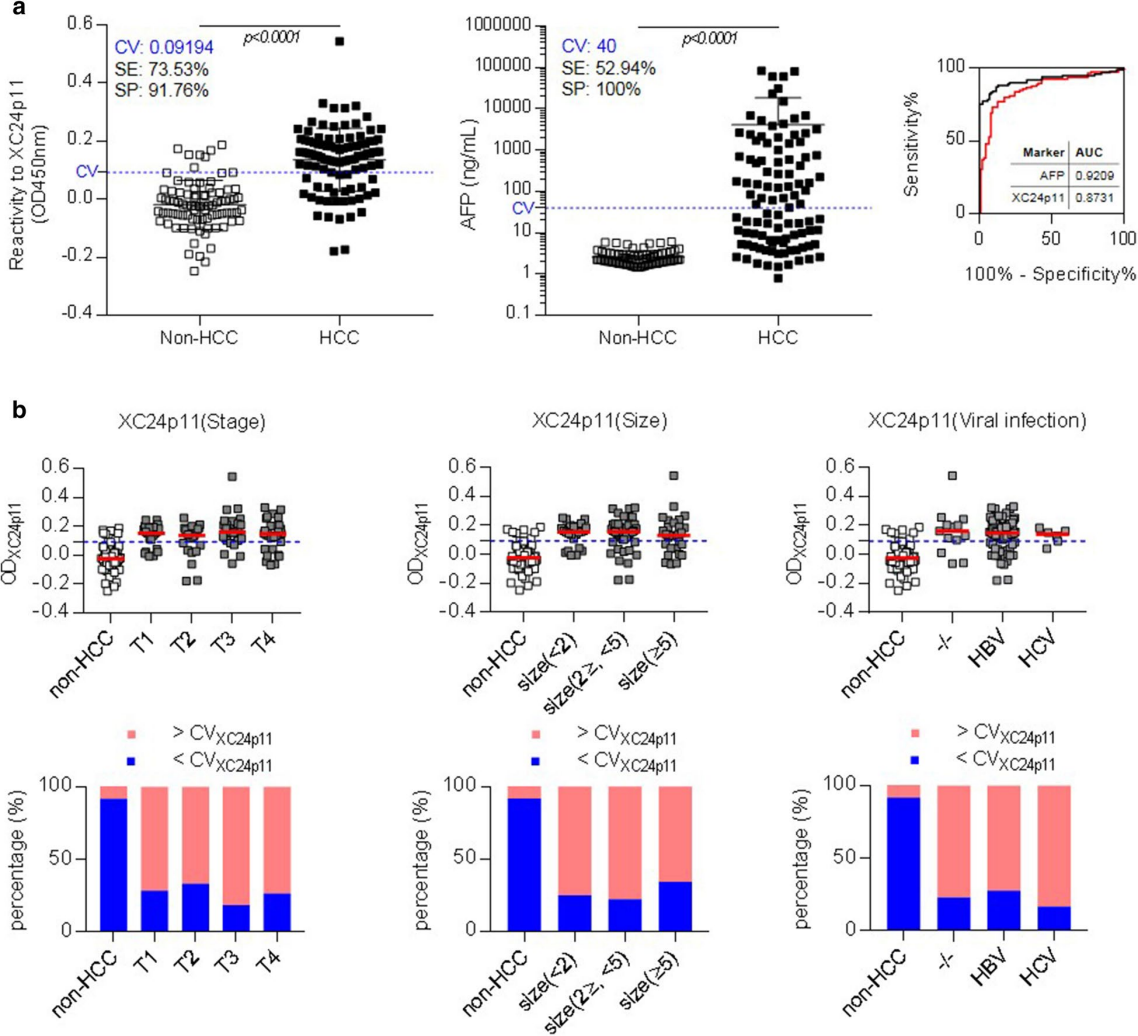
Human serum ELISA using XC24p11-STA to detect anti-SF3B1 autoantibody effectively distinguished between HCC and non-HCC samples. **a** Detection of anti-SF3B1 autoantibody using XC24p11-STA in sera from patients with HCC. The specific binding of autoantibody against XC24p11 epitope was calculated by the difference between the optical density (OD) value of XC24p11-STA ELISA and that of Eph-STA ELISA. Non-HCC (n = 85), HCC (n = 102). AFP was also quantified in the same sample sets using human AFP Quantikine ELISA Kit (R&D systems) and the diagnostic efficiency was evaluated by ROC curve analysis. These experiments were repeated three times and representative results are shown. *CV* cutoff value, *SE* sensitivity, *SP* specificity; **b** Anti-XC24p11-STA ELISA depending on tumor stage, tumor size, and viral infection (HBV or HCV). The upper panel shows the anti-XC24p11 antibody reactivity (OD_XC24p11_) in each serum sample and the lower panel shows the percentage of each group above or below the CV. The clinical information of HCC patients is summarized in Table S1. The dotted line on the plot represents the CV and the short line in red represents the mean value of each group

Using these conditions, ELISA for the detection of human serum autoantibody against SF3B1 was performed. A total of 102 serum samples from HCC patients (Additional file 1: Table S1) and 85 serum samples of healthy control subjects were analyzed, and the reactivity to XC24p11 represented by the difference of antibody reaction between XC24p11-streptavidin (XC24p11-STA) and epitope-free streptavidin (Eph-STA). As shown in Fig. 5a, the reactivity of HCC patient sera against XC24p11 was significantly different from that of the non-HCC control sera, with an AUC value of 0.87 [95% confidence interval (CI) 0.8219–0.9243, p < 0.0001]. The sensitivity of this ELISA was 73.53% and the specificity was 91.76% when the cutoff value (CV) was 0.09194. AFP, a conventional HCC biomarker, was also determined in the same sample set for comparison (Fig. 5a). The CV for the AFP biomarker of 40 ng/ml and ultrasonography were the criteria for evaluation of HCC currently used by clinicians today. AFP detection for HCC diagnosis showed a sensitivity of 52.94% and specificity of 100% when the CV was 40 ng/ml with an AUC value of 0.92 (95% CI 0.8781–0.9637, p < 0.0001). The responses to XC24p11 were analyzed also depending on tumor stage, size, and viral infection status (Fig. 5b). Anti-SF3B1 antibodies were detected in patient’s sera even at the early tumor stage (T1) or in small size tumor (size < 2 cm). Viral infection, which is a major cause of HCC, did not influence anti-SF3B1 autoantibody level. In all subgroups of HCC patients, the portion of the anti-XC24p11 response above the CV was about 70–80%. By contrast, in non-HCC samples, the portion of the anti-XC24p11 response above the CV was about 10%.

### Simultaneous measurement of AFP and anti-SF3B1 autoantibody biomarker in patient serum increased the efficacy of HCC diagnosis

**Fig. 6:**
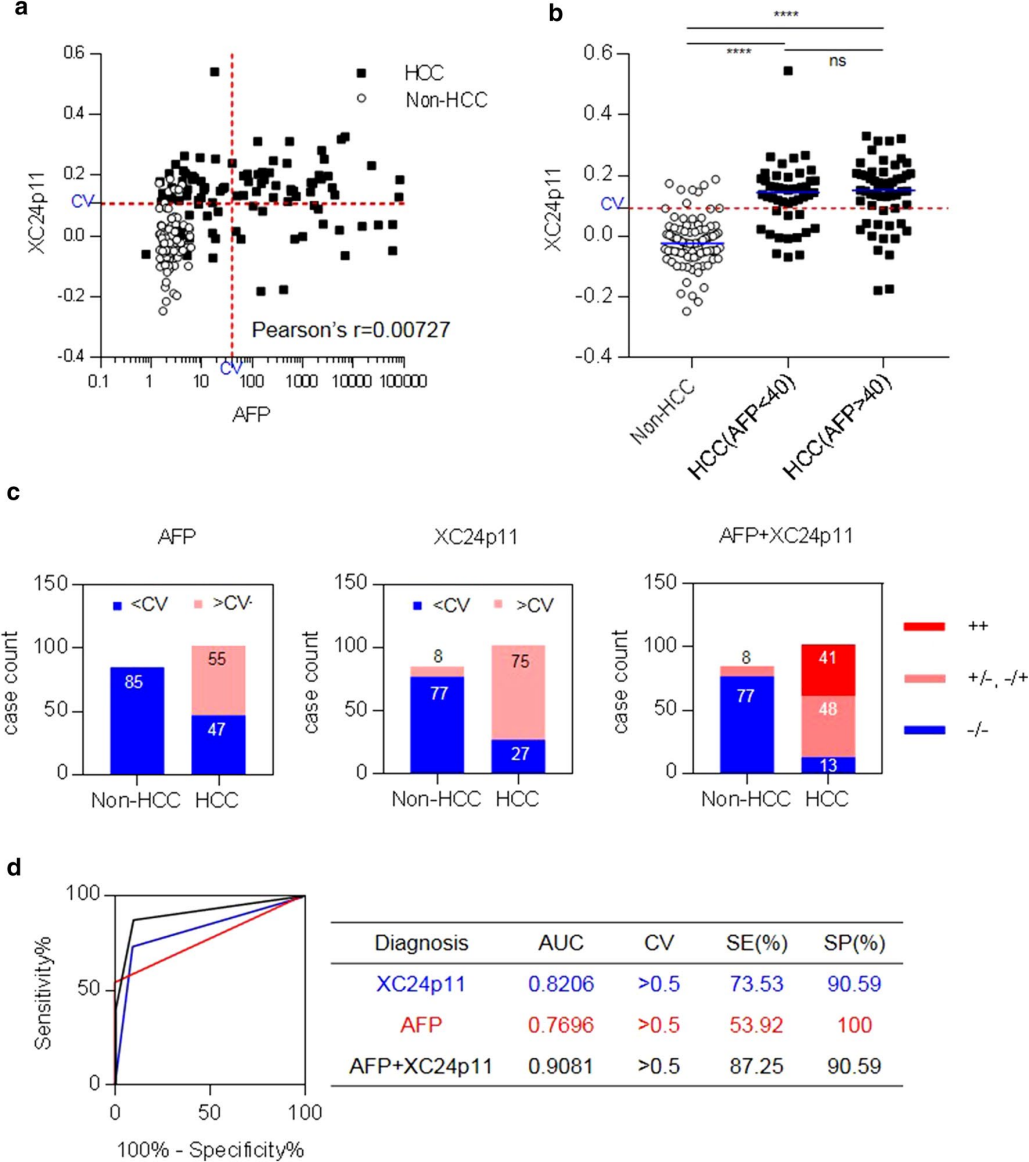
Combination of the HCC biomarker AFP and anti-SF3B1 autoantibody improved the diagnostic efficacy for HCC. **a** Correlation between AFP and anti-SF3B1 autoantibody measured by XC24p11-STA. **b** Anti-SF3B1 autoantibody response depending on AFP levels. **c** Combination analysis of AFP and anti-SF3B1 autoantibody biomarker. The diagnostic values of anti-XC24p11 autoantibody or AFP were indicated as negative (−) or positive (+) according to the value below or above the CV and the subjects were classified into 3 groups [AFP(+)/XC24(+), AFP(+)/XC24(−) or AFP(−)/XC24(+), AFP(−)/XC24(−)]; **d** ROC curve analysis for the combination of AFP and anti-SF3B1 autoantibody biomarker. The diagnostic values of AFP and anti-SF3B1 autoantibody biomarker were arbitrarily indicated as 0 or 1 according to the diagnostic value below or above the CV. The combination of AFP and anti-SF3B1 autoantibody biomarker was performed by adding up the arbitrary diagnostic values of two biomarkers for each subject and then analyzing them again

For decades, AFP has been considered as a gold standard in the diagnosis of liver cancer with high reliability based on long accumulated clinical experiences, even though its diagnostic sensitivity to HCC is low at about 50% [13]. Thus, a novel HCC diagnosis method may be easily acceptable when it complements the defect of AFP test. In this context, we examined the use of the anti-SF3B1 autoantibody test in providing additional information after AFP-based HCC diagnosis. As shown in Fig. 6a, the detection of anti-SF3B1 antibody showed no correlation with AFP level (Pearson’s r = 0.00727). The anti-SF3B1 antibody response pattern of HCC patients was not significantly distinguished between in AFP-low or AFP-high groups [ns (p > 0.05); Fig. 6b]. However, the simultaneous detection of AFP and anti-SF3B1 antibody in serum allowed the subjects to be grouped in detail (Fig. 6c). When the diagnostic values of anti-XC24p11 autoantibody or AFP were indicated as positive (+) or negative (−) according to the value above or below the CV, subjects were classified into 3 groups [AFP(+)/XC24(+), AFP(+)/XC24(−) or AFP(−)/XC24(+), AFP(−)/XC24(−)]. Most of Non-HCC subjects showed AFP(−)/XC24(−) pattern, whereas most of HCC patients showed AFP(+)/XC24(+), AFP(+)/XC24(−) or AFP(−)/XC24(+) pattern. ROC curve analysis of these results showed that although AFP was not detected in half of patients with HCC, the simultaneous detection of AFP and anti-SF3B1 antibodies can discriminate HCC up to 87.25% with AUC value of 0.9081 (95% CI 0.8635–0.9528, p < 0.0001), which is also superior to anti-SF3B1 antibody biomarker detection only (73.53%; Fig. 6d).

## Discussion

SF3B1 is an essential spliceosomal protein, highly conserved during evolution [41]. It is a subunit of splicing factor SF3B, which, together with a second multimeric complex termed SF3A, interacts specifically with the 12S U2 snRNP and converts it into the active 17S form. SF3B1 shows a characteristic intranuclear localization. It is highly concentrated in defined intranuclear structures termed “speckles”, a subnuclear compartment enriched in small ribonucleoprotein particles and various splicing factors. The primary sequence analysis of SF3B1 identified a functional nuclear localization signal of the monopartite type (KRKRR, amino acids 196–200) and speckle-targeting sequence composed of multiple threonine-proline repeats (amino acids 208–513). During mitosis, it also transiently disperses from the nuclear speckles to the cytoplasm. So far, studies on SF3B1 related to cancer have focused on the malfunction of RNA splicing due to the SF3B1 mutation [42]. In this study another aspect of SF3B1 in cancer has been suggested. SF3B1 expression is significantly elevated in liver tissue of HCC and serum anti-SF3B1 autoantibody is identified as a HCC diagnostic biomarker. Although it is a nuclear protein, SF3B1 is secreted into extracellular fluid as a component of exosome. We believe that the increased secretion of SF3B1 caused by cancer progression can stimulate autoantibody production.

Unlike most studies that use patient sera as a source of TA autoantibody biomarkers, we have screened autoantibody biomarkers that respond to human tumor cells in a tumor-model mouse B cell hybridoma pool. Moreover, we suggested a diagnostic ELISA using autoantibody-specific cyclic peptide epitope as a capture antigen. TA autoantigens, which we have identified, including FASN, CK8/18 complex protein and SF3B1, were not easy to prepare as recombinant proteins for use as coating antigens because of their large size or complicated structures; therefore, we screened the minimal antigenic structures against autoantibodies from phage display cyclic peptide library, which simulate only the antigenic structures on target antigen and proposed them as capture antigens for the detection of autoantibody biomarker. Such work was possible because of sufficient supply of antibodies by using monoclonal B cell hybridoma producing TA autoantibodies, a benefit of tumor model mouse study.

The benefit of genetically engineered tumor model mouse for the identification of diagnostic autoantibodies applicable to human patients was also shown by Mao et al. [7]. More importantly, they showed that autoantibody responses against TA autoantigens could discriminate pre-diagnostic sera from non-transgenic mouse control sera, which indicates that transgenic animals could be used to identify cancer-associated autoantibodies present at the earliest stages of the malignant transformation of cancer. Early diagnosis of cancer is critical for effective treatment. Autoantibody biomarker detection methods suitable for the early diagnosis of cancer have not yet been sufficiently developed in spite of its advantages, because prospectively collected human serum samples from cancer patients are rare and not often available for biomarker discovery. Data presented by tumor model mouse implicate that the systematic studies on TA autoantibodies using tumor model mouse can be a breakthrough on the development of early diagnosis of cancer.

In addition to an issue of early diagnosis, precise diagnosis is important. So far, numerous cancer biomarkers have been discovered and applied in appropriately developed diagnostic methods. However, no single biomarker can fully diagnose cancer. In the case of HCC, autoantibody biomarkers can be used to compensate for the flaws of HCC diagnosis using AFP quantification because there is little correlation between AFP and autoantibody detection, as shown in our present results. In this study serum AFP levels were quantified by ELISA and, with currently used cutoff value of 40 ng/ml, analyzed as an HCC diagnostic marker. Using AFP quantification, the sensitivity of HCC diagnosis is about 50% although the specificity is 100%. However, when AFP quantification was combined with the SF3B1 autoantibody test using XC24p11 as a detection antigen, the sensitivity and specificity were increased to 87 and 90%, respectively. Autoantibody biomarkers also have the advantage of being able to be specifically configured for a multiplex diagnosis method to complement each other’s weaknesses of sensitivity and specificity. EarlyCDT, a first commercially available autoantibody detection method, is a multiplex detection method comprising seven autoantibody biomarkers [43, 44]. The study on breast cancer autoantibody biomarkers by Mao et al. also introduced a multiplex autoantibody detection method for diagnosing breast cancer up to 5 autoantibodies [9]. The efficiency of HCC diagnosis using SF3B1 autoantibody as a biomarker is expected to be increased when applied along with other autoantibody biomarkers such as anti-FASN autoantibody, which was previously reported as an HCC biomarker [28]. Collectively, we expect that novel diagnostic methods by autoantibody detection complement the conventional diagnostic methods, which could meet the needs for precision medicine.

## Conclusions

In this study, we identified a novel TA autoantibody, designated as XC24, of which the specific target antigen was identified as splicing factor 3B subunit 1 (SF3B1), and set up an ELISA using cyclic peptide epitope that reacts specifically to XC24, which can be used as a novel in vitro diagnostic method to detect serum anti-SF3B1 autoantibody. We expect that this novel cancer diagnostic method by autoantibody detection complements the conventional diagnostic methods, which could meet the needs for precise medicine.

## Additional files

**Additional file 1: Table S1.** Patient details in validation cohort.

**Additional file 2: Fig. S1.** Verification of XC24 antigen as SF3B1 by immunoprecipitation and western blot analysis: Additional data for Fig. 1f.

**Additional file 3: Fig. S2.** Western blot analysis of intracellular distribution of XC24 antigen or SF3B1: Additional data for Fig. 1h; Total: total cell lysate (40 μg/lane), CYT: cytoplasmic fraction (30 μg/lane), NU: nuclear fraction (10 μg/lane).

**Additional file 4: Fig. S3.** Immunohistochemical staining of live tissues from HCC-model mouse (HBx-Tg, H-*ras-*Tg) with anti-SF3B1 antibody: HBxTg/WT (n = 3), HBxTg/Small Tumor (n = 5), HBxTg/Large Tumor (n = 6), WT (n = 2), H-*ras*-Tg/Tumor (n = 2). DAB intensities of IHC staining were quantified by Image J and plotted in Fig. 2b.

## Abbreviations

AFP: alpha-fetoprotein
AUC: area under the curve
CK8/18: cytokeratin 8/18 complex
CV: cutoff value
ELISA: enzyme-linked immunosorbent assay
FASN: fatty acid synthase
GAPDH: glyceraldehyde-3-phosphate dehydrogenase
HBV: hepatitis B virus
HBx: HBV-encoded X protein
HCC: hepatocellular carcinoma
HRP: horseradish peroxidase
IHC: immunohistochemistry
PCR: polymerase chain reaction
PBS: phosphate-buffered saline
ROC: receiver operating curves
SDS-PAGE: sodium dodecyl sulfate–polyacrylamide gel electrophoresis
SF3B1: splicing factor 3B subunit 1
STA: streptavidin
TBS: tris-buffered saline
