# Supplementary material for: Identification of anti-SF3B1 autoantibody as a diagnostic marker in patients with hepatocellular carcinoma
Source: J Transl Med. 2018 Jun 28;16:177. doi: 10.1186/s12967-018-1546-z (PMC6025833; doi:10.1186/s12967-018-1546-z)
Supplement: Supplementary file 1 — Additional file 1: Table S1. Patient details in validation cohort. [file 12967_2018_1546_MOESM1_ESM.doc]

| **Table S1. Patient details in validation cohort^*^.** | |
| --- | --- |
| HCC patient number | 102 |
| Gender  male/ female, n (%) | 76/ 26 (74.5/ 25.5) |
| Age distribution  yr, Avg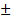SD | 39~83  55.5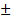10 |
| AFP concentration (ng/mL)  <CV_40_ / CV_40_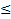, n (%) | 0.8~83000  47/ 55 (46.1/ 53.9) |
| Viral infection  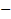/ HBV/ HCV, n (%) | 13/ 83/ 6 (12.7/ 81.4/ 5.9) |
| Tumor stage  T1/ T2/ T3/ T4, n (%) | 21/ 24/ 27/ 30 (20.6/ 23.5/ 26.5/ 29.4) |
| Tumor size  2cm>T/ 2cm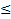T<5cm/ 5cm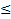T, n (%) | 24/ 49/ 29 (23.5/ 48.1/ 28.4) |
| * Human HCC serum and normal serum samples for this study were provided by the Ajou Human Bio-Resource Bank (AHBB), a member of the National Biobank of Korea. All samples derived from the National Biobank of Korea were obtained with informed consent under institutional review board-approved protocols and the study was approved by Public Institutional Bioethics Committee designated by MOHW ((P01-201409-BS-03; Republic of Korea). Normal human serum samples collected at the Korean Red Cross were exemption from IRB approval which was confirmed by Research Blood Examination Committee. | |
