# Supplementary figures and images for: Identification of anti-SF3B1 autoantibody as a diagnostic marker in patients with hepatocellular carcinoma
Source: J Transl Med. 2018 Jun 28;16:177. doi: 10.1186/s12967-018-1546-z (PMC6025833; doi:10.1186/s12967-018-1546-z)

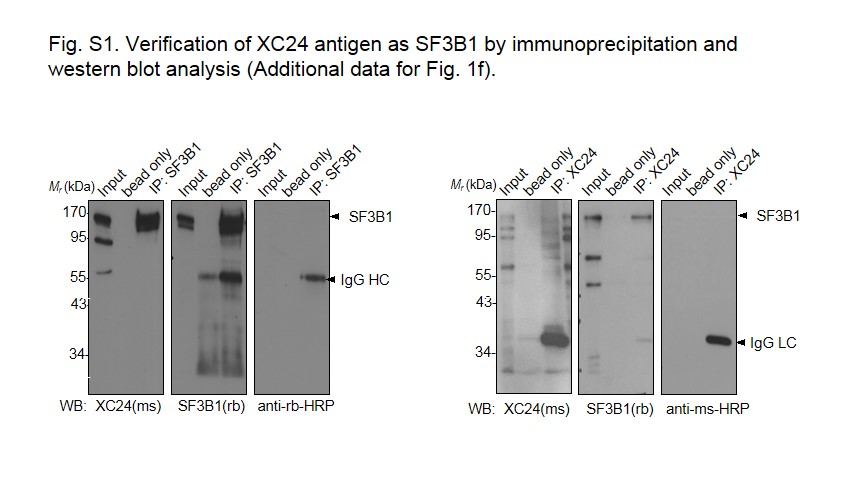

Supplement: Supplementary file 2 — Additional file 2: Fig. S1. Verification of XC24 antigen as SF3B1 by immunoprecipitation and western blot analysis: Additional data for Fig. 1f. [file 12967_2018_1546_MOESM2_ESM.jpg]

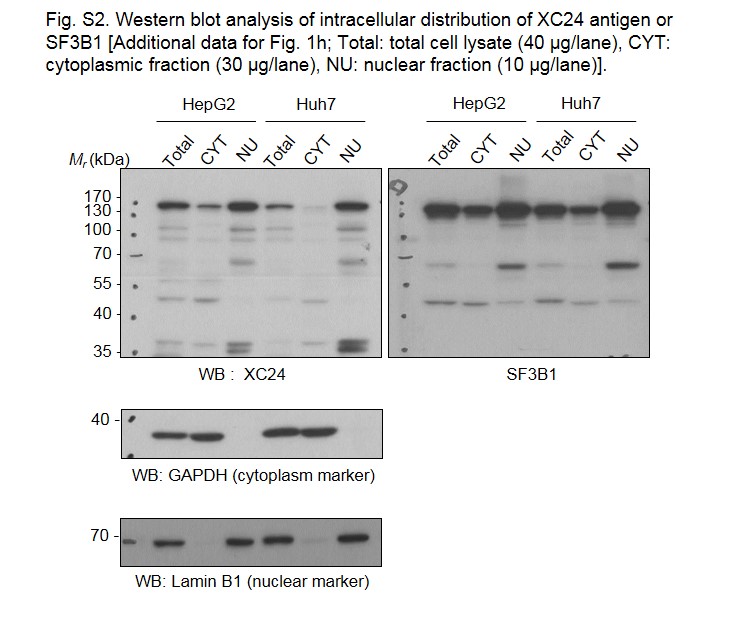

Supplement: Supplementary file 3 — Additional file 3: Fig. S2. Western blot analysis of intracellular distribution of XC24 antigen or SF3B1: Additional data for Fig. 1h; Total: total cell lysate (40 μg/lane), CYT: cytoplasmic fraction (30 μg/lane), NU: nuclear fraction (10 μg/lane). [file 12967_2018_1546_MOESM3_ESM.jpg]

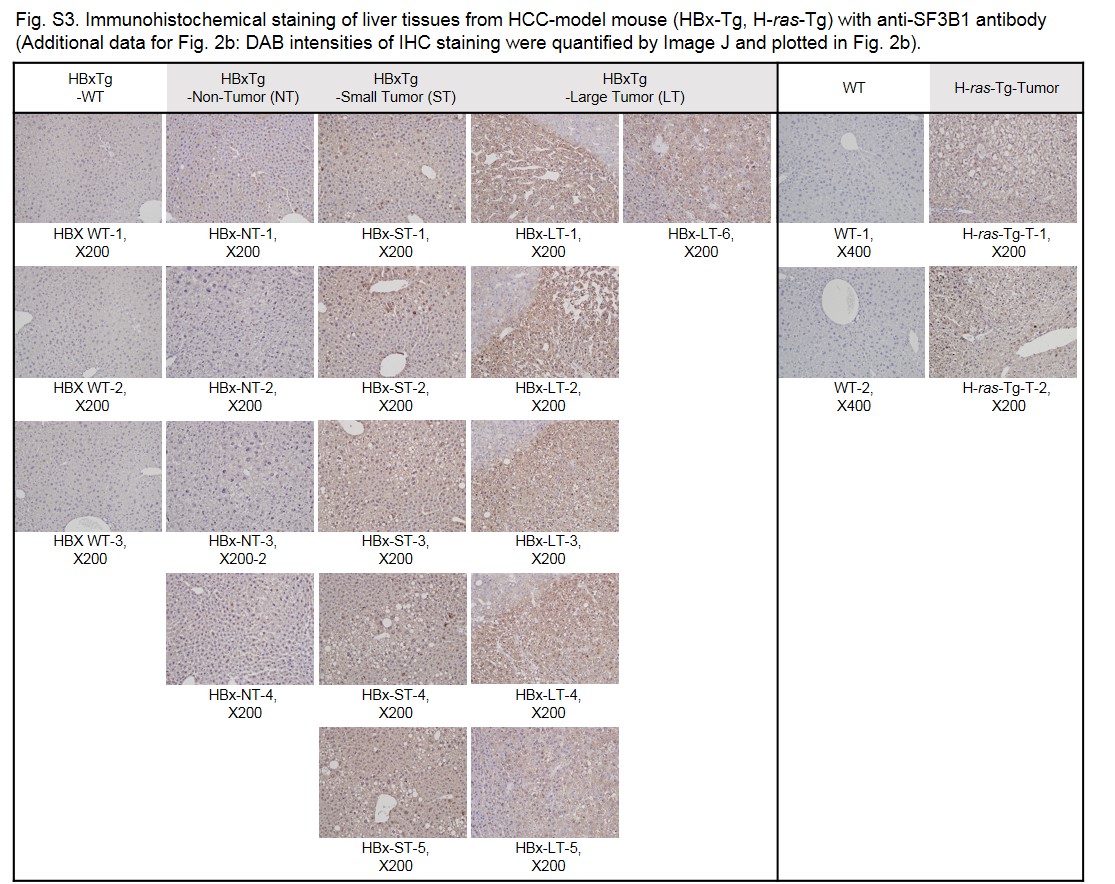

Supplement: Supplementary file 4 — Additional file 4: Fig. S3. Immunohistochemical staining of live tissues from HCC-model mouse (HBx-Tg, H-ras-Tg) with anti-SF3B1 antibody: HBxTg/WT (n = 3), HBxTg/Small Tumor (n = 5), HBxTg/Large Tumor (n = 6), WT (n = 2), H-ras-Tg/Tumor (n = 2). DAB intensities of IHC staining were quantified by Image J and plotted in Fig. 2b. [file 12967_2018_1546_MOESM4_ESM.jpg]
